# Youth suicide crisis: identifying at-risk individuals and prevention strategies

**DOI:** 10.1186/s13034-024-00753-9

**Published:** 2024-05-23

**Authors:** Victor Ajluni, Daniel Amarasinghe

**Affiliations:** Department of Psychiatry and Behavioral Neurosciences, Wayne State University School of Medicine, Detroit, MI USA

## Introduction

In the United States, recent data has shown a disturbing increase in suicide rates among children and adolescents. Recent data has shown a disturbing increase in suicide rates among this demographic. According to the Centers for Disease Control and Prevention (CDC), suicide is now the second leading cause of death for people ages 10–24 years in the United States [1]. The suicide rate for this age group has increased by 56% over the past decade, with Black youth having the largest increase in suicide rate of 78% [2].

The AAP-AACAP-CHA Declaration of a National Emergency in Child and Adolescent Mental Health is a joint statement by the American Academy of Pediatrics, the American Academy of Child and Adolescent Psychiatry, and the Children’s Hospital Association regarding this ongoing crisis [3]. The joint statement highlights the importance of recognizing suicide risk and the need for increased funding to address the issue. In addition, it emphasizes that teachers, physicians, and other professionals who work with children and adolescents have a crucial role to play in suicide prevention. There is a great need for these professionals to be able to recognize the warning signs and risk factors for suicide and take appropriate action to intervene and connect young people with mental health resources. In addition to raising awareness about suicide risk, the declaration also calls for increased access to mental health care services and investment in research and public health initiatives aimed at preventing and treating mental health disorders in children and adolescents.

### Risk factors and warning signs

Access to firearms increases the likelihood of death by suicide for children and adolescents more than fourfold due to its increased lethality, with 90% of child and adolescent suicides by gun involving firearms from the victim’s own home or that of a relative [4]. When controlling differences in the rate of youth suicide attempts across states, it was found that household gun ownership was positively associated with the overall youth suicide rate. For 10% increase in household gun ownership, the youth suicide rate increased by 26.9% [5].

Across age groups, a past suicide attempt is the strongest predictor of subsequent attempts, with up to a 12-fold increase in children, a 4.3-fold increase in adolescent and 5.4-fold increase in young adults [6]. Although some studies suggest that suicidal ideation is associated with a higher risk of suicide and is far more prevalent than attempted suicides, a considerable percentage of children and adolescents that commit suicide have no prior suicide attempts [7, 8]. In fact, a metanalysis found that 3% of high-risk patients discharged from a psychiatric hospital could be expected to commit suicide within a year, and that 60% of the patients that do commit suicide would have likely been categorized as low risk. The study also found that there was no single risk factor or combination of risk factors that was strongly associated with risk of suicide in the year after discharge [9]. Although the United States Preventive Services Task Force (USPSTF) recommends screening children 12 and older for depression, and ages 8 and older for anxiety, they state that there is insufficient data to recommend for or against screening for suicidal risk in all youth, which brings into question the utility of screening for suicidal ideation [10]. One study found that 66% of people that committed suicide within 30 days of seeing a healthcare professional denied suicidal ideation, and that 50% of this group committed suicide within 2 days after being seen [11].

On the contrary, some argue that screening for suicidal risk in clinical settings such as primary care and emergency departments can identify people at elevated risk for suicide who would otherwise be missed if they did not present primarily for behavioral health concerns [12]. Proponents of universal screening of youth for suicidal risk also state that the risks associated with screening are minimal, and people that screen positive for suicidal ideation or behavior would benefit from a mental health referral. However, it is important to note that suicidal ideation may be less frequent in Black youth prior to a suicide attempt, which highlights the importance of developing complementary methods to self-reporting tools for the assessment of suicide risk [12].

Substance use, particularly marijuana use, has been associated with increased suicide-related mortality in both adolescents and adults. One study found that states with medical marijuana legalization or recreational marijuana legalization had increased suicide-related mortality in 14–16-year-old people, with higher rates of suicide in states with recreational marijuana legalization compared to states with medical marijuana legalization (IRR = 1.14, 95% CI: 1.00-1.30) and states without marijuana laws (IRR = 1.09, 95% CI: 1.00-1.20) [13].

Some factors that contribute to suicidal ideation and self-injurious behavior but not necessarily completed suicides are socioeconomic stressors, trauma, adverse childhood events, social media use and bullying. Pre-adolescent children that have safety concerns are more likely to attempt suicide, which highlights the impact of socioeconomic stressors such as low-income and poor social support on increased suicide risk. A history of mental illness is also associated with a higher likelihood of a suicide attempt across all age groups [14].

People with exposure to traumatic experiences or adverse childhood experiences are at an increased risk of attempting suicide, with up to a 2 to 5-fold increase. Adverse childhood experiences include childhood abuse, witnessing domestic violence, parent separation or divorce, cohabitation with household members with mental illness, and cohabitation with household members that engage in substance use or criminal activity [14]. A growing concern is the use of social media to bully or harass peers, with 57% of bullied persons reaching the clinical threshold for PTSD on a reporting scale [15]. It was also found that in children and adolescents that reported using social media had significantly greater predicted odds of self-injurious behaviors at the time of admission to the emergency department [16].

Some warning signs for suicide include [17]:

1. Showing signs of depression, anxiety, or other mental health issues.
2. Talking or writing about suicide, death, or dying.
3. Withdrawing from friends and family.
4. Showing signs of extreme mood swings or sudden personality changes.
5. Displaying reckless or risky behavior.
6. Engaging in self-harm or self-destructive behavior.
7. Losing interest in activities they used to enjoy.
8. Having difficulty sleeping or sleeping too much.
9. Feeling hopeless or trapped in a situation.
10. Giving away prized possessions.

### Prevention and intervention

Numerous studies globally have shown that school-based, community-based and primary-care centered interventions across a range of settings and populations lead to a significant reduction in both suicide risk and suicide attempts in adolescents [18].

Regarding school-based prevention programs, a meta-analysis of 12 studies showed that post-primary school-based suicide prevention programs had a significant decrease on the adolescent suicidal thoughts and behaviors, with a number needed to treat of 55 for suicidal ideations, and 20 for suicide attempts. Overall, that post-primary school-based suicide prevention programs led to a 13–15% reduction in suicidal ideation and a 28–34% reduction in suicide attempts among 33,155 adolescents attending 329 schools [19].

Primary care providers are the front-line for community intervention, which involve identifying and screening for people at risk for suicide. One such community-based intervention program involved the following: primary care provider training on identifying and appropriately referring people with depression, a media and public relations campaign, funding for community facilitators, and additional support for both the patients and their families. The cost-effective program included training in suicide risk assessment and management and access to resources like crisis hotlines and mental health services. The study found that the 2-year intervention was associated with a 32.4% decrease in attempted and completed suicides [20].

Aside from group-based interventions, some individual interventions to reduce the risk of suicide include dialectical behavioral therapy, psychoeducation for parents regarding suicide risk and lethal means restriction. Pharmacological treatment such as lithium and clozapine has been found to reduce the risk of suicide in adults, but additional studies in children and adolescents are needed [21].

## Conclusion

The AAP-AACAP-CHA Declaration of a National Emergency in Child and Adolescent Mental Health is a critical call to action for addressing the mental health crisis facing young people in the United States, particularly in considering the alarming rise in suicide rates. The declaration emphasizes the importance of recognizing suicide risk and taking appropriate action to intervene and connect young people with mental health resources. It also calls for increased access to mental health care services and investment in research and public health initiatives aimed at preventing and treating mental health disorders in children and adolescents. With the right interventions and resources in place, it has been shown that we can work towards reducing suicide rates among young people and providing them with the support they need to thrive. As a society, it is our responsibility to prioritize the mental health of our youth and address this urgent issue.

## Bottom line

Access to firearms is one of the biggest risk factors for youth suicide. Suicidal ideation is not a reliable predictor of future suicide attempts or completed suicide, although it may be useful in identifying those that would be otherwise missed if they did not present originally for mental health concerns. Marijuana use has been associated with an increased rate of suicide in states with recreational marijuana legalization or medical marijuana legalization. In addition to previous self-injurious thoughts and behavior, adverse childhood experiences, bullying and excessive social media usage are associated with an increase in suicidal ideation but not necessarily an increase in suicide risk. School-based, community-based and primary-care centered interventions are associated with a significant reduction in both suicide risk and suicide attempts in adolescents.

## Data Availability

Not applicable.
